# The Efficacy and Safety of Fractional CO_2_ Laser Combined with Topical Type A Botulinum Toxin for Facial Rejuvenation: A Randomized Controlled Split-Face Study

**DOI:** 10.1155/2016/3853754

**Published:** 2016-02-22

**Authors:** Jie Zhu, Xi Ji, Min Li, Xiao-e Chen, Juan Liu, Jia-an Zhang, Dan Luo, Bing-rong Zhou

**Affiliations:** ^1^Department of Dermatology, The First Affiliated Hospital of Nanjing Medical University, Nanjing 210029, China; ^2^Department of Dermatology, The First Affiliated Hospital of Nanjing University of TCM, Nanjing, Jiangsu 210029, China; ^3^Department of Dermatology, Nanjing Maternal and Child Health Hospital Affiliated to Nanjing Medical University, Nanjing, Jiangsu 210029, China; ^4^Department of Dermatology, Nanjing Children's Hospital of Nanjing Medical University, Nanjing, Jiangsu 210029, China; ^5^Department of Dermatology and Venereology, Nanjing Jingdu Hospital, Nanjing, Jiangsu 210029, China

## Abstract

*Objective*. We evaluated synergistic efficacy and safety of combined topical application of Botulinum Toxin Type A (BTX-A) with fractional CO_2_ laser for facial rejuvenation.* Methods*. Twenty female subjects were included for this split-face comparative study. One side of each subject's cheek was treated with fractional CO_2_ plus saline solution, and the other side was treated with fractional CO_2_ laser plus topical application of BTX-A. Patients received one session of treatment and evaluations were done at baseline, one, four, and twelve weeks after treatment. The outcome assessments included subjective satisfaction scale; blinded clinical assessment; and the biophysical parameters of roughness, elasticity, skin hydration, transepidermal water loss (TEWL), and the erythema and melanin index.* Results*. BTX-A combined with fractional CO_2_ laser sides showed higher physician's global assessment score, subject satisfaction score, roughness, skin hydration, and skin elasticity compared to that of fractional CO_2_ plus saline solution side at 12 weeks after treatment. TEWL and erythema and melanin index showed no significant differences between two sides at baseline, one, four, and twelve weeks after treatment.* Conclusion*. Topical application of BTX-A could enhance the rejuvenation effect of fractional CO_2_ laser.

## 1. Introduction

Fractional CO_2_ laser has now been considered as the gold standard for skin rejuvenation [1]. Nevertheless, when it is used for facial rejuvenation, it often requires multiple therapy sessions which will be a kind of psychological and economic burden for the patients. Besides that, fractional CO_2_ laser is still reported to have a variety of complications, such as postinflammatory hyperpigmentation, prolonged erythema, skin swelling, infection, and scarring [2, 3]. How to reduce adverse reactions of fractional CO_2_ laser, while enhancing its rejuvenation effect, has become one hot topic in dermatological research field.

Botulinum Toxin Type A (BTX-A) is one of seven neurotoxins produced by* Clostridium botulinum*. BTX-A can result in muscle fiber atrophy and subsequent clinical flaccid paralysis [4]. Consequently, its cosmetic use by subdermal injections in treating wrinkles induced by muscle hyperactivity is widespread [5]. Some physicians have observed a face-lifting effect after intradermal injection of BTX-A to the mid and lower face [6, 7]; however the underlying mechanisms still remain unclear. In 2012, Oh et al. studied the* in vitro* effects of BTX-A on normal fibroblasts and found that BTX-A has a significant effect in increasing the level of collagen production and downregulating its degradation [8]. In 2014, our research group further confirmed the direct antiphotoaging potential of BTX-A in UVB-induced premature senescence of human dermal fibroblasts* in vitro* through decreasing senescence-related proteins [9]. Whether BTX-A shows its direct skin rejuvenation effects has not been studied* in vivo* as yet.

Recent studies have also reported the transepidermal delivery of drugs, stem cells secreted factors, and even bone marrow mesenchymal stem cells using a fractional ablative laser [10–12]. According to the results of previous literatures, we can speculate that BTX-A can pass through the micropores created by fractional CO_2_ laser and penetrate into the dermis, thereby executing various biological effects. In present 12-week clinical study, BTX-A solution was topically administrated following fractional CO_2_ laser; we then focused on the effects of BTX-A on the efficacy and adverse effects after fractional CO_2_ laser.

## 2. Material and Methods

### 2.1. Subjects

This was a single-center prospective pilot study. All subjects provided written informed consent, and this study was approved by the Institutional Review Board of the First Affiliated Hospital of Nanjing Medical University. Twenty female subjects with Fitzpatrick phototypes III and IV were enrolled in the study after signing an informed consent form. Patients were aged 21 to 53 (mean age 35.6). Exclusion criteria were keloids and hypertrophic scars, cancer lesions, warts or skin infections in the area to be treated, viral herpes infections during the previous 6 months, collagen disease, and autoimmune disease. Individuals who had taken systemic isotretinoin, BTX-A injection, or any facial rejuvenation procedures during the previous 12 months, had used nonreabsorbable fillers, were undergoing treatment with antineoplastics, corticosteroids, or anticoagulants, or were diabetic, pregnant, or breastfeeding were excluded.

### 2.2. Treatment Protocol

A topical anesthetic cream (2.5% lidocaine and 2.5% prilocaine; Tsinghua Ziguang Co., Beijing, China) was applied for 30 minutes before treatment and then completely removed. A fractional CO_2_ laser (Acupulse, Lumenis, Inc., Santa Clara, CA, USA) was used for the fractional laser treatment. We treat both cheeks' area of subjects with DeepFX microscanner handpiece of the fractional ultrapulsed CO_2_ laser. The laser parameters were set: single pass, 0.12 mm spot size, pulse energy 10 mJ, density 5%, pulse size 10 × 10 mm, and repetition rate 300 Hz. The peak power and beam width of the fractional ultrapulsed CO_2_ laser are 200 watt and 50–80 *μ*s, respectively. Ten milliliters of BTX-A solution (diluted in saline solution, concentration at 5 *μ*/mL) was topically applied onto the fractional laser-treated sites of one randomly selected facial side for one hour, while saline solution was applied to fractional laser-treated sites of the another face side for one hour. The outcome assessments included the subjective satisfaction scale, improvement score according to blinded investigators (using standardized photography), and biophysical measurements. Measurements were conducted at baseline, one, four, and twelve weeks after the treatment. Subjects were permitted to apply their usual skin care products throughout the study. It was requested that subjects do not alter their usual skincare routine during the study period.

### 2.3. Outcome Assessment

#### 2.3.1. Subjective Satisfaction Scale

Subjects completed a self-assessment questionnaire and rated their improvement on a scale from 0 (aggravated) to 4 (much improved). After treatment, subjects were asked to grade their intraprocedure pain on a 10 cm visual analog scale (VAS), with the end points designated as 0 (no pain) and 10 (the worst pain imaginable). The investigator subjectively graded edema after treatment on a scale from 0 to 4 (0 = absent, 1 = trace, 2 = slight, 3 = moderate, and 4 = prominent). The duration of erythema and crusting was investigated through interviews. Any adverse events and complications were recorded at the time of each treatment and at the follow-up visit.

### 2.4. Blinded Clinical Assessment

Standardized photographs were obtained at baseline and 1 month after the last treatment. Standardized close-up photographs were taken using a Visia photo stand (Canfield Imaging Systems, Fairfield, NJ) mounted with a high resolution digital camera (Canon EOS-40D, Canon Corp., Tokyo, Japan). Two dermatologists who were blinded to subject treatment group evaluated the serial photographs independently and performed clinical assessments on fine wrinkles, coarse wrinkles, roughness, mottled hyperpigmentation, laxity, and skin tone using a well-established grading scale of 0 ≤ 25% (minimal), 1 = 26–50% (fair), 2 = 51–75% (good), 3 = 75–90% (excellent), and 4 = 91–100% (clear) improvement. Average improvement scores were calculated as the mean of the grading scales of all categories.

### 2.5. Biophysical Evaluations

The parameters assessed were* in vivo* erythema, melanin, transepidermal water loss (TEWL), elasticity, skin surface roughness, and hydration, which were measured with respective probes (Courage and Khazaka Electronic GmbH, Cologne, Germany). All measurements were taken after subjects had undergone an acclimatization period of at least 20 minutes in an air-conditioned room under standardized conditions (22–25°C, 50% humidity). Each measurement was performed on the left and right cheeks of each subject.

### 2.6. Statistical Analysis

The results were analyzed with the paired *t*-test using SPSS 15.0 software (SPSS, Inc., Chicago, IL). *P* < 0.05 was considered to be significant.

## 3. Results

### 3.1. Objective Clinical Assessment

**Figure 1:**
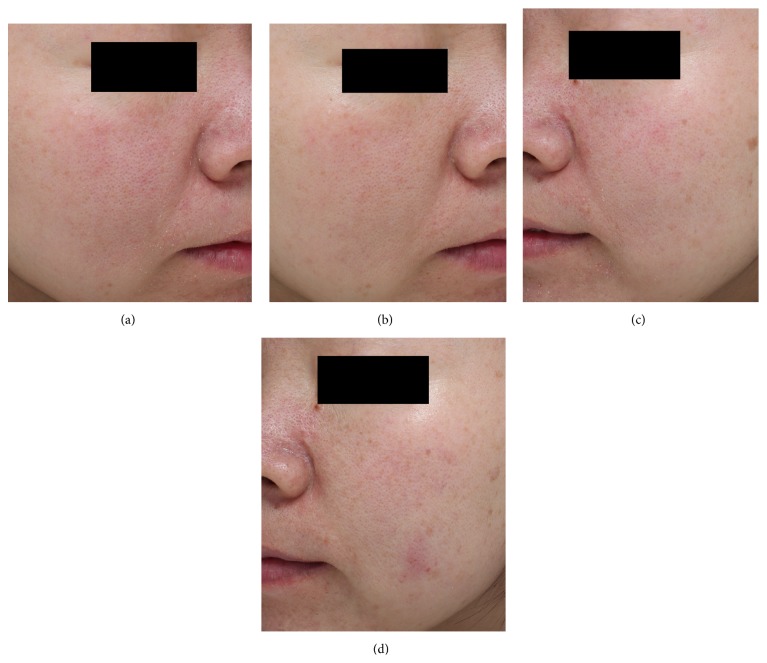
Representative clinical photographs: (a, c) baseline, (b) 12 weeks after the BTX-A + fractionated CO_2_ laser treatment, and (d) 12 weeks after the saline solution + fractionated CO_2_ laser treatment.

Figure 1 shows representative clinical manifestations of both treatment sides taken at indicated time points. During the evaluation periods, two blinded evaluators scores increased successively in both sides. Twelve weeks after the treatment, BTX-A side objective clinical assessment score value was 3.40 ± 0.42 which was significantly higher than that of saline solution side (2.70 ± 0.43) (*P* < 0.05).

### 3.2. Subjective Satisfaction Scale

During the evaluation periods, the subjective satisfaction scores in both sides increased successively. Twelve weeks after the treatment, BTX-A side subjective satisfaction score value was 3.40 ± 0.53 which was significantly higher than that of saline solution side (2.70 ± 0.47) (*P* < 0.05).

### 3.3. Biophysical Analysis

#### 3.3.1. Erythema Index (EI) and Melanin Index (MI)

**Figure 2:**
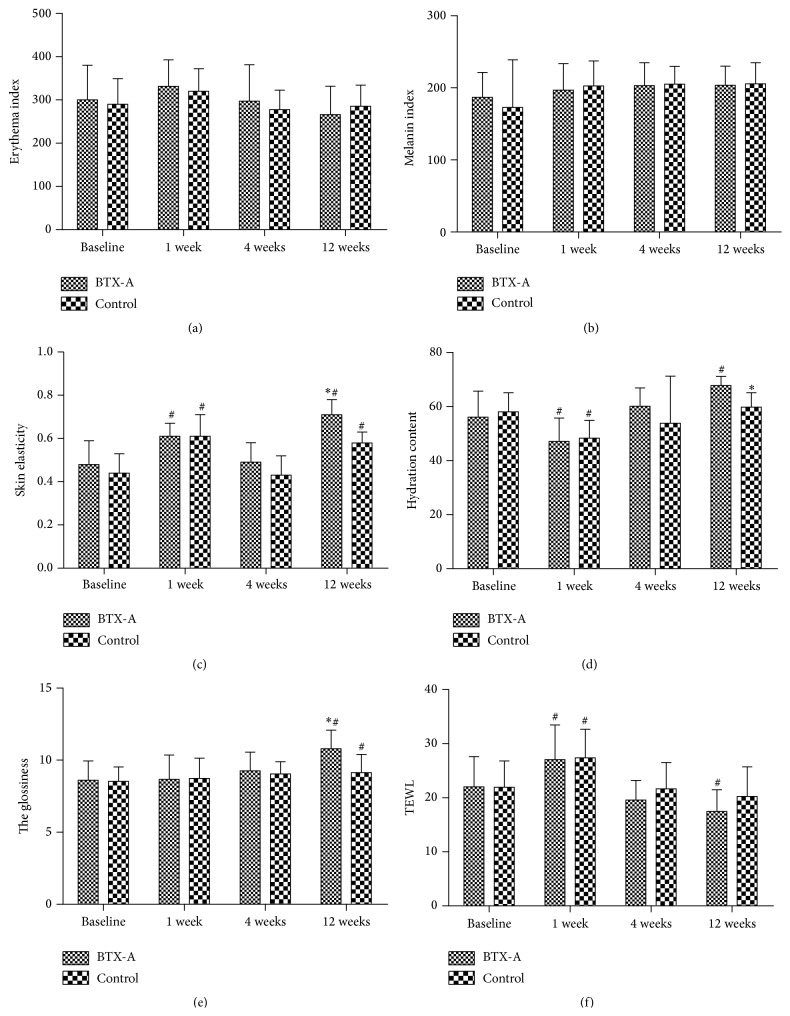
Skin measurements by biophysical evaluation methods were taken at baseline, 1 week, 4 weeks, and 12 weeks after treatment on both sides. (a) Objective measurement of erythema index, (b) melanin index, (c) overall elasticity (*R*2), (d) hydration, (e) glossy, and (f) TEWL. ^*∗*^ *P* < 0.05 compared with control side at same time point; ^#^ *P* < 0.05 compared with baseline.

As shown in Figures 2(a) and 2(b), during the study, the average value of EI and MI in both sides achieved the peak value at one week after the treatment, while decreasing to nearly baseline four weeks after treatment. However, the differences between the two sides were not statistically significant at any given point of time after the treatment.

### 3.4. Elasticity

As shown in Figure 2(c), one week after the treatment, the skin elasticity of both sides was higher than that prior to the treatment, then decreasing to nearly baseline four weeks after the treatment. Twelve weeks after the treatment, the skin elasticity in BTX-A side increased and was higher than that of baseline; however, it remained nearly unchanged in saline solution treated side and the difference of elasticity between the two sides was statistically significant (*P* > 0.05).

### 3.5. TEWL

TEWL was as one of the noninvasive indices to evaluate the skin barrier integrity or function. As shown in Figure 2(f), the value of TEWL increased at one week after the treatment and then rapidly decreased to baseline at four weeks after the treatment. In BTX-A side, the average values even showed a lower level than baseline and are statistically significantly lower than that of saline solution side at twelve weeks after treatment (*P* < 0.05).

### 3.6. Hydration

Hydration content was also considered as one of the indices to evaluate the skin barrier. As shown in Figure 2(d), hydration increased from 56.08 ± 9.59 to 67.76 ± 3.38 in BTX-A side. The hydration values in BTX-A side shown were significantly higher than that of saline solution side at twelve weeks after treatment (*P* < 0.05).

### 3.7. Skin Surface Roughness

As shown in Figure 2(e), skin surface roughness increased significantly (from 8.53 ± 1.0 to 9.13 ± 1.26) in saline solution side and more significantly increased in BTX-A side (from 8.61 ± 1.33 to 10.79 ± 1.29). The differences between the two sides were noted at twelve weeks after treatment (*P* < 0.05).

### 3.8. Comparison of Adverse Reactions

No serious or persistent side effects occurred during the course of study, and none of the subjects withdrew from the study because of any adverse event. No hypopigmentation or hypertrophic scarring was observed in any subject throughout the study period. As shown in Table 1, there was no significant difference of pain score, edema score, duration of crust, and duration of erythema between the two sides.

## 4. Discussion

Regarding erythema and melanin index, there are no significant differences between BTX-A side and saline solution control side up to 12 weeks after laser treatment in all subjects. Several reports demonstrated that BTX-A can be used for the treatment of facial erythema and flushing [13–15]. The clinical results were substantiated with corresponding decreases in cutaneous blood flow, as measured using laser Doppler flowmetry [16]. Although BTX-A cannot reduce erythema index after laser treatment, it at least did not induce excessive angiogenesis as shown in the present study. Besides that, there are not any significant differences between the two sides in regard to the pain score, edema score, duration of crusting, and duration of erythema, which verify that adjuvant BTX-A treatment may not help promote the recovery of laser-damaged skin and decrease downtime.

Although an increase in the level of TEWL and a decrease in the hydration content in the stratum corneum one week after the treatment were the signs of impaired skin barrier function, these two indices in both sides recovered to the baseline levels at four weeks after the treatment. Interestingly, at twelve weeks after the treatment, the TEWL was significantly lower, while hydration was significantly higher in BTX-A side than that of saline solution side. These results suggested that although BTX-A did not have an impact on skin recovery, it improves skin barrier function quality after laser treatment. A recent research indicated that the skin barrier function was enhanced after the injection of a new variety of BTX [17]. Besides that, Oh et al. also found that BTX-A could increase the viability of skin fibroblasts, suggesting its role in promoting cell growth and wound healing [8]. Moreover, some researchers have also proven that BTX-A could accelerate the healing process of several kinds of skin wounds [18–20], suggesting BTX-A may activate the proliferation and migration of human skin cells which are important for skin barrier function maintenance. In one word, our results in the present* in vivo* study further confirmed that better skin barrier function can be achieved by BTX-A topical treatment.

There have been many studies which have suggested that fractional CO_2_ laser can significantly improve skin texture and wrinkles [21–23]. Similar to previous reports, the subjective satisfaction scores of skin elasticity and texture were significantly better than control sides. Besides that, we also confirmed by biophysical analysis that the skin elasticity and skin surface roughness in BTX-A sides were also higher than those of control sides 12 weeks after treatment. These results suggested that topical application of BTX-A can significantly enhance the facial rejuvenation effect of fractional CO_2_ laser. Several* in vivo* histopathological researches have confirmed that depth of the microthermal zones of fractionated CO_2_ laser, whose settings are similar to the present study, was limited to the middermis [24–26]. Skin permeation of small-molecule drugs, macromolecules, and nanoparticles mediated by a fractional carbon dioxide laser mostly target the skin dermis [27, 28]. In this consideration, we speculate that BTX-A solution used in the present study may take its action mainly on dermis rather than muscle fibers alone. Besides that, the best facial rejuvenation effect of topical BTX-A appeared 12 weeks after laser treatment which further supports our hypothesis. In our previous study, we found BTX-A has positive effects on photoaged fibroblasts* in vitro* by increasing collagen production, decreasing collagen degradation, and stimulating cell proliferation via decreasing senescence-related proteins [9]. However, up to date, it remains unclear with which cellular signal pathway BTX-A acts as an antiaging molecule. Its exact functional mechanisms deserve further basic in-depth researches.

One major shortcoming of this study is that it is merely a small sample prospective clinical study. A larger sample randomized controlled clinical trials need to verify the results of this study. To the best of our knowledge, our study is first to report the efficacy and safety of topical BTX-A markedly enhancing the facial rejuvenation effect of fractional CO_2_ laser. We suggest that topical BTX-A can be used as an adjuvant therapy after fractional CO_2_ laser. Its exact mechanism of action and how to optimize its clinical effects are still worth further researches.

## Figures and Tables

**Table 1:** Comparison of adverse events on both sides after treatment **(** $\bar{x}\pm S$ **)**.

Group	Pain score	Edema score	Duration of incrustation (d)	Duration of erythema (d)
BTX-A treatment	5.03 ± 0.81	1.73 ± 0.58	7.43 ± 2.27	20.29 ± 1.75
Saline solution treatment	4.95 ± 1.03	1.97 ± 0.72	7.23 ± 2.64	20.45 ± 1.33

*Note*. No significant differences were observed on both cheeks (*P* > 0.05).
